# Mouthwash with Active Oxygen (blue®m) Reduces Postoperative Inflammation and Pain

**DOI:** 10.1155/2021/5535807

**Published:** 2021-05-31

**Authors:** Bruna Marca Mattei, Soraia A. W. Imanishi, Grasieli de Oliveira Ramos, Paloma Santos de Campos, Suyany Gabriely Weiss, Tatiana Miranda Deliberador

**Affiliations:** ^1^Universidade do Oeste de Santa Catarina (UNOESC), Joaçaba, Santa Catarina, Brazil; ^2^Universidade Federal do Rio Grande do Sul (UFRGS), Porto Alegre, Rio Grande do Sul, Brazil; ^3^School of Health Sciences, Department of Dentistry, Universidade Positivo, Curitiba, Paraná, Brazil; ^4^Latin American Institute of Dental Research and Education (ILAPEO), Curitiba, Paraná, Brazil

## Abstract

The aim of this case series was to evaluate the effects of blue®m mouthwash on oral surgical wounds. Eleven patients underwent bilateral preprosthetic surgery and were instructed to apply the product only to the right side of the surgery. In this way, the right side corresponds to the test side and the left side (place without applying any type of solution) to the control side. After seven days of using the product (3 times a day), the following parameters were evaluated by means of a visual analogue scale: pain, changes in taste, and acceptance by the patient. Then, the level of tissue inflammation was assessed, by the number of pixels, using ImageJ® software. The main results show that the blue®m mouthwash was widely accepted by patients, reducing their pain. The number of inflammation pixels was lower on the test side (*p* < 0.05), indicating improved healing. It is suggested that blue®m mouthwash positively influences tissue healing reducing pain and the postsurgical inflammatory process; however, randomized clinical trials should be done to prove this clinical observation.

## 1. Introduction

Chlorhexidine (CHX) is considered the gold standard in the antiseptic treatment of the oral mucosa, due to its broad antibacterial spectrum [1, 2]; it is recommended in postsurgical follow-ups, since mechanical hygiene control could cause trauma to the surgical wound, rupture of the suture, and loss of the ability to keep tissues in position [3, 4]. However, studies have shown that CHX has a cytotoxic effect on gingival fibroblasts [5, 6], gingival epithelial cells [7], periodontal ligament cells [8], cultured alveolar bone cells [9], and osteoblastic cells [10]. It has also been demonstrated to interfere with tissue regeneration processes [10]. From the search for a mouthwash that minimized the undesirable effects with the use of CHX, new lines of research are needed, among them, the treatment with active oxygen [11].

A team of dental surgeons led by Dr. Peter Blijdorp in the Netherlands developed a product based on active oxygen (blue®m) with the intention of putting all the desirable properties of mouthwashes in just one product. blue®m mouthwash has in its composition sodium perborate, the enzyme glucose oxidase derived from honey, xylitol, and lactoferrin [12]. Applications in dentistry vary from use in inflammation of gums and ulcers, peri-implantitis, and after oral surgery to a substitute for hypochlorite sodium during irrigation of root canals [13]. Inclusion in everyday hygienic oral care of toothpaste and mouthwash blue®m reduced the severity of inflammatory changes and improved the hygienic condition of the oral cavity in cardiology patients suffering from periodontal disease [14]. A randomized controlled clinical trial showed that toothpaste containing active oxygen and lactoferrin has comparable antiplaque and antigingivitis efficacies with triclosan-containing toothpaste [15]. Previous in vitro studies from the team have demonstrated that blue®m at higher concentrations provided inhibitory halo of Porphyromonas gingivalis similar to chlorhexidine digluconate [16] and human keratinocyte cell line demonstrated a greater proliferation rate when exposed to lower concentrations of blue®m mouthwash [17]. Nevertheless, more studies are needed to evaluate patient experience and the effects on wound healing.

Wound healing is an extremely complex process, which requires a variety of cells to increase its metabolic activity, resulting in oxygen demand [18]. The application of topical oxygen to the wound has been shown to promote healing through several processes, including antibacterial activities, neovascularization, collagen production, epithelialization, phagocytosis (encompassing microorganisms, cells, or debris by macrophages or neutrophils), and degradation of necrotic tissue wound [19]. Topical Oral Oxygen Therapy (OOT) is aimed at accelerating the healing process by ensuring neovascularization, removing toxins, stimulating the formation of new blood cells, increasing the production of stem cells, and eradicating bacteria [20]. OOT with blue®m can be a substitute for CHX in postsurgical care, not showing the disadvantages observed with the use of CHX, especially in relation to the cytotoxic effect on gingival cells [12].

The local use of active oxygen as support for wound healing and tissue regeneration may be a tendency to replace the use of CHX, but it inspires parsimony, due to the small number of clinical monitoring cases reported or clinical trials in the literature. It is hypothesized that the use of the mouthwash will improve healing thus reducing postoperative pain. Hence, the aim of this case series is to evaluate the effects of blue®m mouthwash (OOT) in postoperative oral surgery care.

## 2. Case Series Report

### 2.1. Ethical Aspects

This study was approved by the Ethics and Research Committee on Human Beings at UNOESC under the number 2.031.509 CAAE: 65808817.3.0000.5367. Each patient received and signed an informed consent form and an image use term. The work was conducted in compliance with the Declaration of Helsinki.

### 2.2. Inclusion Criteria

A sample of 14 patients of both sexes, aged between 54 and 74 years old, who underwent preprosthetic surgeries at Universidade do Oeste de Santa Catarina (UNOESC), Joaçaba, São Paulo, Brazil, was selected.

### 2.3. Exclusion Criteria

The exclusion criteria involved patients that used drugs that could directly interfere with the tissue response, such as anticoagulants and antiplatelet agents, patients who have been on antibiotic or anti-inflammatory therapy in the past three months, diabetics, smokers, and individuals who have reported a history of hypersensitivity to any mouthwash. Also, irradiated patients, pregnant women, and patients who presented lesions such as candidiasis or lichen were considered exclusion criteria.

### 2.4. Study Design

The patients were submitted to preprosthetic surgery to remove hyperplasia of the maxillary posterior and anterior ridge. Infiltrative anesthesia was performed in the anterior border region using 4% articaine with epinephrine 1 : 100,000. Two parallel linear incisions were made, along the upper or lower alveolar ridge, extending along the excessive fibrous tissue over the ridge. The two incisions were found at the base (deepest region), forming a wedge of fibrous tissue. After making the incisions, the fibrous tissue was carefully removed with the aid of a Molt detacher. The continuous scalloped suture was performed with 4-0 silk thread. The surgeries were performed by different operators.

After preprosthetic surgery, each patient was prescribed acetaminophen 750 mg and received 50 ml of the test antiseptic (blue®m mouthwash) together with 15 units of flexible rods with cotton tips. In the postoperative period, the patient was instructed to apply the solution over the entire length of the region, only on the right side up to the midline (below the nose), with the aid of flexible rods three times a day (upon waking up, after lunch, and before bedtime), for a week. The left side served as a control. Patients were instructed not to rinse their mouths with water or other mouthwashes and neither to eat for 30 minutes after applying the solution.

### 2.5. Follow-Up

**Figure 1:**
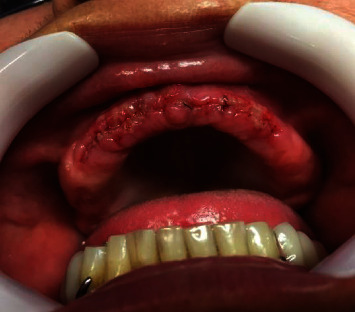
7-day postoperative period. Image used for analyzing pixels.

The postoperative evaluation was performed 7 days after the surgery, at the time of suture removal (Figure 1). An intraoral physical examination was performed to assess the healing of the surgical wound. Pain was measured using the visual analogue scale (VAS). Patients were asked about changes in taste, the occurrence of adverse effects, and whether they would use the antiseptic again. After removing the suture, the operated area was dried with gauze and photographic records were made. All recordings were performed with a cell phone camera (iPhone 6, Apple®, California, USA) with the aid of retractors (Lip Retractor Expandex—Indusbello®, Paraná, Brazil). The patient was positioned lying down for all sites operated on the upper arch and seated for all surgical sites on the lower arch. All data collections were performed by the same researcher.

### 2.6. Photographic Analysis

The photos of the healing surgical wound were analyzed using the ImageJ® program, developed by Wayne Rasband of the Research Services Branch, National Institute of Mental Health (Bethesda, Maryland, USA), which is a domain Java image processor and image analyzer software inspired in NIH Image for the Apple Macintosh. The repertoire of functions of this software can be expanded by means of several ready-made plugins, available on the Internet, or by developing new ones.

**Figure 2:**
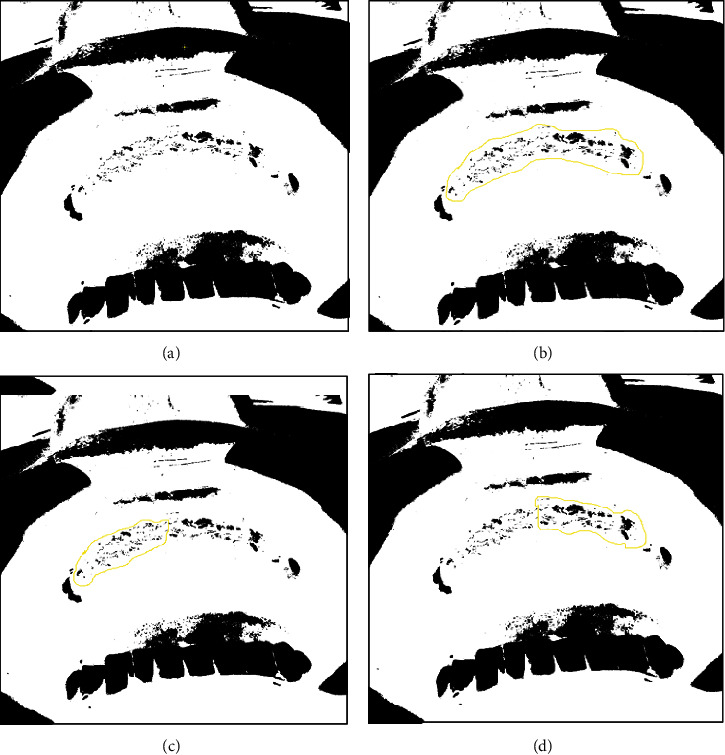
Analysis of images using the ImageJ® program for red pixels. (a) Image obtained after the appropriate adjustment of the red color scale; the software turns the area of interest to black and the other areas to white. (b) Segmentation of the total area of interest to obtain the total amount of red pixels. (c) Segmentation of the right area (test side) to obtain red pixels. (d) Segmentation of the left region (control side) to obtain red pixels in this area.

Areas of inflammation (red), considered a less healed oral tissue, and areas of healthy tissue (pink), considered with a better healing tissue, were selected. A saturation pattern was set for all images to equalize the contrast by 0.5%. Through the threshold color plugin available on the Internet, the selection of colors given in pixel shapes was carried out. The area of interest was defined, whether covered with healthy tissue or granulation/inflammation. The number of red pixels (Figure 2) and pink pixels was obtained after appropriate adjustment of the scale, where the software turns the areas that satisfy the chosen pattern to black and the other areas to white.

### 2.7. Statistical Analysis

All analyses were performed with a significance level of 0.05 using the Bioestat® Program version 5.3. Pain values (test side and control side) were compared using the ANOVA test. The healing images were subjected to the ANOVA test of paired samples.

## 3. Results

There were 14 patients in the study, 8 were women and 11 were men, and 81% of them were over 60 years old. Three were excluded: one because he was unable to attend the follow-up on the 7th day to remove the suture and for the photographic record and the other two because they had used antiseptic throughout the length of the surgery. Therefore, the study evaluated 11 patients.

**Figure 3:**
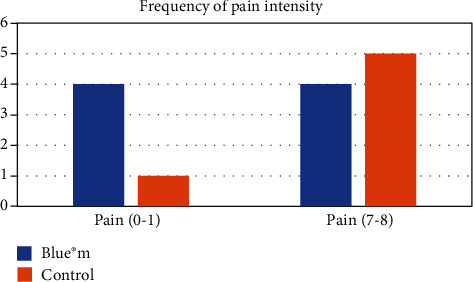
Frequency of pain by stratification of pain intensity.

**Figure 4:**
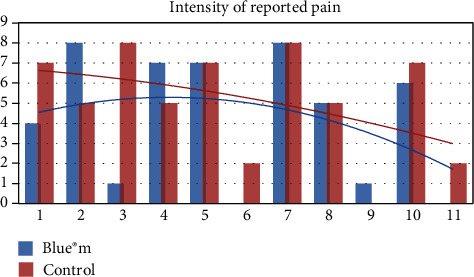
Intensity of reported pain between the control and test sides. ANOVA, *p* = 0.0233.

Regarding the minimum pain measured by patients (categorized by pain intensity numbers 0 and 1), it was more often reported on the test side than on the control side (Figure 3). The most severe pain (categorized by pain intensity numbers 7 and 8) was reported more frequently on the control side (*p* < 0.05), as shown in Figure 4. It is possible to see in the adjusted curve of the graph that the average pain intensity is lower for the test side.

There were no patient-reported changes in taste, nor was there any adverse effect. Everyone replied that they would use the product again.

**Figure 5:**
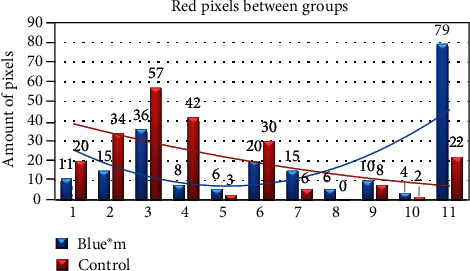
Comparison of the number of red pixels between the control and test sides. ANOVA: two-paired samples. *p* < 0.05.

The control and test sides analyzed by the ImageJ® program showed statistical significance (*p* < 0.05) in relation to the number of red pixels (Figure 5) but did not show statistical significance (*p* > 0.05) when analyzed in relation to the number of pink pixels. More than 90% of the patients had a lower index of red pixels on the test side, while only 72% of patients presented with a lower index of red pixels on the control side.

## 4. Discussion

Postoperative pain and the presence of inflammation are common symptoms after oral surgeries. In this series of cases, we opted to prescribe for patients the topical application of a new mouthwash (blue®m) that has a slow release of oxygen, with the hypothesis that, on the side that the product was applied, the patient would feel less pain and have a lesser inflammatory process. Our results showed that the hypothesis can be accepted. The mouthwash effects seem to reduce postoperative pain and inflammation.

Some people have difficulty controlling plaque build-up by only conventional tooth cleaning. Oral surgery usually requires the use of chlorhexidine mouthwashes, in addition to conventional tooth cleaning, to avoid injuries, drop of sutures, or even patient's inability. Chlorhexidine is an effective chemical method of plaque control, used for the treatment of several periodontal conditions, such as gingivitis [21, 22]. However, the use of chlorhexidine is associated with a variety of local side effects such as brown staining of the teeth and oral tissues [23], altered taste perception [23–25], supragingival calculus accumulation [25, 26], and oral mucosal lesions [23, 25]. Thus, the search for mouthwash as effective as chlorhexidine with fewer side effects has become relevant. blue®m products have oxygen as an active ingredient, and it was demonstrated that toothpaste containing active oxygen and lactoferrin has comparable antiplaque and antigingivitis efficacies with triclosan-containing toothpaste [15]. It is also known that oxygen application is involved in several stages of the healing process, such as reepithelialization, angiogenesis, and collagen synthesis [20]. Therefore, the present case series is aimed at evaluating the effects of blue®m mouthwash in postoperative oral surgery care.

This split-mouth model using test and control sides in the same patient was based on many intraoral studies in humans and also in animal studies. This methodological aspect was similar to that used by Laureano Filho et al. [27] and Marlière et al. [28], which allowed qualifying the patient as a control group for himself. In this way, it was possible to control individual variations (eating habits, metabolism, hygiene, and personal behavior) in addition to variations in the surgical procedure itself (operator, duration, type of anesthetic, and technique). However, it is still necessary to consider a bias in our study, which is the possible interference of prostheses in the postsurgical period. We believe that in two cases (in which the most severe pain occurred precisely on the test side), it was not due to the ineffectiveness of the antiseptic but because perhaps there was interference from the prosthesis. Still, in relation to this bias, there are reports that the resilient material used as a temporary reshaper also affected the healing of soft tissues [29].

Despite the presence of the product having reached a statistical difference, the pain intensities reported between the test and control sides should be more discrepant. One answer to this inconsistency would be the inappropriate choice of the method of collecting pain measurement in elderly patients. According to Gold and Roberto [30], the Visual Analogue Scale (VAS) requires a higher level of cognitive function; therefore, it may be inappropriate for patients with low levels of education and with cognitive changes. Considering that our patients really had difficulty in quantifying the intensity of pain, we corroborate that, for elderly people with a low level of formal education, perhaps the best method of measuring pain is the face scale, as proposed by the same authors.

It is known that there are many difficulties in performing clinical analysis of tissue healing. So, in this series of cases, we chose to use a methodology that was able to make the comparison between the test and control sides using an objective program (ImageJ®). This methodology had already been carried out in medical studies and is now beginning to be introduced in dentistry [31–33].

The analysis of healing by ImageJ® revealed that sites treated with blue®m showed a higher frequency of less inflamed sites (fewer red pixels). However, there was no difference when analyzing the number of more healed sites (pink pixels). The work of Pansani et al. [34] clarifies this issue. It has been reported that the individual's age factor alters the synthesis and proliferation of fibroblasts but has no influence on their inflammatory activity. Therefore, fibroblasts from older individuals maintain the capacity of the inflammatory response similar to what occurs in fibroblasts from young individuals. Thus, in the studied sample, regardless of age, the product appears to have reduced inflammation. In the improvement of healing, no difference was observed between the sides; one of the justifications is the time period of only 7 days of the analyses.

Photographic monitoring is used to monitor and illustrate the different degrees of severity of skin lesions. Benson et al. [35] used photographic images to monitor dental treatment using the Pro-Plus® software. In this manuscript, the authors were concerned with reducing the reflection produced in photography with the aid of specific devices, demonstrating that it influences the results of image analysis, and therefore, there is a need for standardization in image capture. In our study, the photographic records were made only with ambient light and avoiding shadows, which may explain the lack of pixel difference between the test and the control sides.

The results of the present study prove to be promising. Therefore, studies on new mouthwash alternatives are necessary to reduce any harmful effect on oral tissues, especially during the postsurgical healing phase, aiding in healing and decreasing the morbidity of oral surgeries.

## 5. Conclusion

Within the limits of this study, blue®m mouthwash seems to reduce postoperative pain as well as clinical signs of inflammation.

## Data Availability

The data used to support the findings of this study are included within the article.

## References

[1] Loe H., Rindom Schiott C. (1970). The effect of mouthrinses and topical application of chlorhexidine on the development of dental plaque and gingivitis in man. *Journal of Periodontology*.

[2] Jenkins S., Addy M., Newcombe R. G. (1994). Dose response of chlorhexidine against plaque and comparison with triclosan. *Journal of Clinical Periodontology*.

[3] Batista F. C., Batista J., Eraldo L., Fronza B. R. (2002). Características microscópicas de superfície e biocompatibilidade dos fios de sutura mais utilizados na cirurgia bucal. *Revista Brasileira de Cirurgia e Implantodontia*.

[4] Aranega A., Okamoto T., Jardim Junior E. G. (1999). Clorexidina e fio de sutura: influência da antissepsia com clorexidina sobre a contaminação bacteriana em suturas com fio de poliéster. Estudo microbiológico e histomorfológico em ratos. *Revista Gaúcha de Odontologia*.

[5] Boisnic S., Ben Slama L., Branchet-Gumila M. C., Watts M., D’Arros G. (2006). Wound healing effect of Eludril^®^ in a model of human gingival mucosa. *Revue de Stomatologie et de Chirurgie Maxillo-faciale*.

[6] Mariotti A. J., Rumpf D. A. (1999). Chlorhexidine-induced changes to human gingival fibroblast collagen and non-collagen protein production. *Journal of Periodontology*.

[7] Babich H., Wurzburger B. J., Rubin Y. L., Sinensky M. C., Blau L. (1995). An *in vitro* study on the cytotoxicity of chlorhexidine digluconate to human gingival cells. *Cell Biology and Toxicology*.

[8] Chang Y. C., Huang F. M., Tai K. W., Chou M. Y. (2001). The effect of sodium hypochlorite and chlorhexidine on cultured human periodontal ligament cells. *Oral Surgery, Oral Medicine, Oral Pathology, Oral Radiology, and Endodontics*.

[9] Cabral C. T., Fernandes M. H. (2007). *In vitro* comparison of chlorhexidine and povidone-iodine on the long-term proliferation and functional activity of human alveolar bone cells. *Clinical Oral Investigations*.

[10] Gianelli M., Chellini F., Margheri M., Tonelli P., Tani A. (2008). Effect of chlorohexidine digluconate on different cell types: a molecular and ultrastructural investigation. *Toxicology In Vitro*.

[11] (1985). *Oxigen enriched water and oral oxygen terapy*.

[12] April 2020, https://www.swallowdental.co.uk/catalogues/Bluem-Compendium.pdf

[13] KRIJNEN P. W. (2011). Healing oxygen: healing action applied in dentistry. https://static.webshopapp.com/shops/035143/files/056054158/dutch-journal-dentistry-tandartspraktijk-healing-o.pdf?_ga=1.179714342.323417379.1487907128.

[14] Makeeva I. M., Tambovtseva N. V. (2014). Applying toothpaste and mouthwash BLUEM in complex oral care in patients with coronary heart disease. *Stomatologiia (Mosk)*.

[15] Cunha E. J., Auersvald C. M., Deliberador T. M. (2019). Effects of active oxygen toothpaste in supragingival biofilm reduction: a randomized controlled clinical trial. *International Journal of Dentistry*.

[16] Deliberador T. M., Weiss S. G., Rychuv F. (2020). Comparative analysis *in vitro* of the application of blue oral gel versus chlorhexidine on *Porphyromonas gingivalis*: a pilot study. *Advances in Microbiology*.

[17] Mattei B., Imanishi S., Ramos G., Campos P., Weiss S., Deliberador T. (2020). Mouthwash with active oxygen (blue) induces keratinocytes proliferation. *Open Journal of Stomatology*.

[18] Schreml S., Szeimies R. M., Prantl L., Karrer S., Landthaler M., Babilas P. (2010). Oxygen in acute and chronic wound healing. *The British Journal of Dermatology*.

[19] Knighton D. R., Hunt T. K., Scheuenstuhl H., Halliday B. J., Werb Z., Banda M. J. (1983). Oxygen tension regulates the expression of angiogenesis factor by macrophages. *Science*.

[20] Eisenbud D. E. (2012). Oxygen in wound healing: nutrient, antibiotic, signaling molecule, and therapeutic agent. *Clinics in Plastic Surgery*.

[21] James P., Worthington H. V., Parnell C. (2017). Chlorhexidine mouthrinse as an adjunctive treatment for gingival health. *Cochrane Database of Systematic Reviews*.

[22] da Costa L. F. N. P., Amaral C. D. S. F., Barbirato D. D. S., Leão A. T. T., Fogacci M. F. (2017). Chlorhexidine mouthwash as an adjunct to mechanical therapy in chronic periodontitis: a meta-analysis. *Journal of the American Dental Association (1939)*.

[23] Addy M. (1986). Chlorhexidine compared with other locally delivered antimicrobials. A short review. *Journal of Clinical Periodontology*.

[24] Marinone M. G., Savoldi E. (2000). Chlorhexidine and taste. Influence of mouthwashes concentration and of rinsing time. *Minerva Stomatologica*.

[25] Strydonck D. A., Slot D. E., Velden U., Weijden F. (2012). Effect of a chlorhexidine mouthrinse on plaque, gingival inflammation and staining in gingivitis patients: a systematic review. *Journal of Clinical Periodontology*.

[26] Mandel I. D. (1994). Antimicrobial mouthrinses: overview and update. *Journal of the American Dental Association (1939)*.

[27] Laureano Filho J. R., Camargo I. B., Firmo A. C. B., Oliveira e Silva E. D. (2008). Evaluation of laser therapy in edema, pain and trismus reductipon after removal of inferior third molars:preliminary results. *Revista de Cirurgia e Traumatologia Buco Maxilo Facial*.

[28] Marlière D. A. A., Lanini L. F., Bittencourt T. C., Assis N. M. S. P. (2015). Combination therapeutic dexamethasone and meloxicam in inflammatory controlafter third molars surgery. *Revista de Cirurgia e Traumatologia Buco Maxilo Facial*.

[29] Chaves C., Vergani C. E., Thomas D. (2014). Biological effects of soft denture reline materials on L929 cells in vitro. *Journal of Tissue Engineering*.

[30] Gold D. T., Roberto K. A. (2000). Correlates and consequences of chronic pain in older adults. *Geriatric Nursing*.

[31] Damante C. A., Greghi S. L., Sant'ana A. C., Passanezi E. (2004). Clinical evaluation of the effects of low-intensity laser (GaAlAs) on wound healing after gingivoplasty in humans. *Journal of Applied Oral Science*.

[32] Schara R., Sersa I., Skaleric U. (2009). T1 relaxation time and magnetic resonance imaging of inflamed gingival tissue. *Dentomaxillofacial Radiology*.

[33] Cáceres M., Oyarzun A., Smith P. C. (2014). Defective wound-healing in aging gingival tissue. *Journal of Dental Research*.

[34] Pansani T. N., Basso F. G., Soares D. G., Hebling J., Costa C. A. S. (2016). Functional differences in gingival fibroblasts obtained from young and elderly individuals. *Brazilian Dental Journal*.

[35] Benson P. E., Shah A. A., Willmot D. R. (2005). Measurement of white lesions surrounding orthodontic brackets: captured slides vs digital camera images. *The Angle Orthodontist*.

